# NDRG2 ablation reprograms metastatic cancer cells towards glutamine dependence *via* the induction of ASCT2

**DOI:** 10.7150/ijbs.48066

**Published:** 2020-10-16

**Authors:** Mingchao Ding, Xin Bu, Zhehao Li, Haokun Xu, Lin Feng, Junbi Hu, Xinxin Wei, Jiwei Gao, Yanyan Tao, Bolei Cai, Yanpu Liu, Xuan Qu, Liangliang Shen

**Affiliations:** 1 The State Key Laboratory of Cancer Biology, Department of Biochemistry and Molecular Biology, The Fourth Military Medical University, Xi'an, 710032, China.; 2 State Key Laboratory of Military Stomatology &National Clinical Research Center for Oral Diseases&Shaanxi Clinical Research Center for Oral Diseases, Department of Oral and Maxillofacial Surgery, School of Stomatology, The Fourth Military Medical University, No. 145 Changle Xi Road, Xi'an, 710032, China.; 3 Department of Oral and Maxillofacial Surgery, Hospital of Stomatology, Jiamusi University, Jiamusi, 154002, China.; 4 State Key Laboratory of Military Stomatology &National Clinical Research Center for Oral Diseases & Shaanxi International Joint Research Center for Oral Diseases, Department of Oral Anatomy and Physiology and TMD, the Fourth Military Medical University, Xi'an 710032, China.; 5 Shaanxi University of Chinese Medicine, Xianyang, 712046, China.; 6 Department of Gastroenterology, the First Affiliated Hospital of Xi'an Jiaotong University, Xi'an, 710061, China.; 7 Department of General Surgery, Tangdu Hospital, Air Force Medical University, Xi'an, 710032, China.; 8 Xi'an Peihua University, Xi'an, 710125, China.

**Keywords:** NDRG2, ASCT2, c-Myc, glutaminolysis, mucoepidermoid carcinoma, EMT

## Abstract

**Background:** Metastasis is the most common cause of lethal outcome in various types of cancers. Although the cell proliferation related metabolism rewiring has been well characterized, less is known about the association of metabolic changes with tumor metastasis. Herein, we demonstrate that metastatic tumor obtained a mesenchymal phenotype, which is obtained by the loss of tumor suppressor NDRG2 triggered metabolic switch to glutamine metabolism.

**Methods:** mRNA-seq and gene expression profile analysis were performed to define the differential gene expressions in primary MEC1 and metastatic MC3 cells and the downstream pathways of NDRG2. NDRG2 regulation of Fbw7-dependent c-Myc stability were determined by immunoprecipitation and protein half-life assay. Luciferase reporter and ChIP assays were used to determine the roles of Akt and c-Myc in mediating NDRG2-dependent regulation of ASCT2 in in both tumor and NDRG2-knockout MEF cells. Finally, the effect of the NDRG2/Akt/c-Myc/ASCT2 signaling on glutaminolysis and tumor metastasis were evaluated by functional experiments and clinical samples.

**Results:** Based on the gene expression profile analysis, we identified metastatic tumor cells acquired the mesenchymal-like characteristics and displayed the increased dependency on glutamine utilization. Further, the gain of NDRG2 function blocked epithelial-mesenchymal transition (EMT) and glutaminolysis, potentially through suppression of glutamine transporter ASCT2 expression. The ASCT2 restoration reversed NDRG2 inhibitory effect on EMT program and tumor metastasis. Mechanistic study indicates that NDRG2 promoted Fbw7-dependent c-Myc degradation by inhibiting Akt activation, and subsequently decreased c-Myc-mediated ASCT2 transcription, in both tumor and NDRG2-knockout MEF cells. Supporting the biological significance, the reciprocal relationship between NDRG2 and ASCT2 were observed in multiple types of tumor tissues, and associated with tumor malignancy.

**Conclusions:** NDRG2-dependent repression of ASCT2 presumably is the predominant route by which NDRG2 rewires glutaminolysis and blocks metastatic tumor survival. Targeting glutaminolytic pathway may provide a new strategy for the treatment of metastatic tumors.

## Introduction

Mucoepidermoid carcinoma (MEC) is one of the most common primary oral and maxillofacial malignant salivary gland tumors 1. The primary low/intermediate-grade MECs are always comforting and predict better outcomes, whereas patients with the high-grade MECs generally carried a lymph node or distant metastasis, which is the strongest prognostic factor of treatment failure 2, 3.

Glucose is converted into lactate even in the presence of adequate oxygen supply, particularly in malignant cancer cells 4, 5. The increased glycolysis is closely related to cell proliferation, metastasis, drug resistance, and poor prognosis in numerous cancers 4. Previous studies demonstrated that cancer cells with high glycolytic rate are commonly dependent on glutamine utilization, which can catabolize glutamine to generate ATP and maintain the mitochondrial function for metabolism through glutaminolysis 6-8. Especially, sustained growth and survival of most cancer cells rely upon a metabolic rewiring, characterized by an enhanced glycolytic flux and a stimulated utilization of glutamine by reductive carboxylation 9, 10. In the process of mitochondrial glutaminolysis, glutamine is initiated by amino acid transporter ASCT2 (alanine-serine-cysteine transporter 2; SLC1A5), and then enter the TCA cycle through being converted to glutamate by glutaminase GLS1/2 and subsequently to α-ketoglutarate (α-KG), which can provide energy and biological macromolecular materials for tumor cell survival 11.

ASCT2 is a Na^+^ dependent neutral amino acid transporter belonging to the SLC1 family, which catalyzes a sodium-dependent obligatory antiport among glutamine and other neutral amino acids 12. Numerous studies demonstrated that ASCT2 mediates the entry of glutamine to the glutaminolysis pathway to generate building blocks and energy for anabolic purposes 13, 14. ASCT2 expression increases in highly proliferative cells, such as inflammatory, stem cells and various cancers, to fulfill the augmented glutamine demand, which has generated interest in its candidacy as a pharmacological target for new anticancer drugs 12. In mammals, two different phosphate-activated glutaminase isoforms were identified: GLS1 (kidney-type) and GLS2 (liver-type), which are encoded by separate genes on different chromosomes 15. GLS1 is more broadly expressed in human normal tissues and cancers, while GLS2 expression is restricted primarily to the liver and brain 16. Thus GLS1 is thought to play a more critical role in during cancer development. Though the key modulators driving metabolic reprogramming have been addressed, such as the activations of c-Myc and HIF-1 17, 18, whether and how the metabolic-related genetic alterations contribute to tumor metastasis and aggressive phenotype generation are still largely unchartered.

Tumor metastasis requires epithelial-mesenchymal transition (EMT) to change cell morphology and extracellular matrix components and facilitate cell migration and invasion 19. During EMT, tumor cells change from an epithelioid morphology to mesenchymal cell morphology, accompany with the diminished epithelial marker E-cadherin and increased mesenchymal marker Vimentin and N-cadherin, which are operated by EMT-related transcription factor Snail, Slug, et al. 20. After executing the EMT program, epithelial-derived cancer cells acquire high-grade malignant traits, including dedifferentiation, invasiveness, and the resistance to apoptosis and chemotherapy, which results in the metastatic dissemination from primary tumors 21. Various oncogenic signaling pathways are involved in this regulation, such as TGF-β 22, Wnt/β-catenin 23, PI3K/AKT 24. Emerging lines of evidence suggest that the dysregulation of metabolic pathways are associated with EMT induction. The enhanced glycolytic rate was observed in mesenchymal cells 25, 26, and the key enzymes related to glycolysis serve important roles in EMT and tumor metastasis, including PKM2 27, FBP1 28 and LDHA 29. Though the role of glutaminolysis in the regulations of tumor metastasis has been investigated, such as the activation of GLS1 or ASCT2 in metastatic tumors 30, 31, more work is needed to fully elucidate the glutaminolysis function in this process.

NDRG2 (N-Myc downstream regulated gene 2), belonging to N-Myc downstream gene family, is a tumor suppressor gene firstly identified in our lab 32. Emerging lines of evidence show that NDRG2 serves an important role in regulating the proliferation, differentiation, apoptosis and metastasis of multiple types of malignant tumors 33, 34. In our previous work, we isolated a highly metastatic clone MC3, which was from the third passage metastatic lesion of mucoepidermoid carcinoma MEC1 cells in nude mice 2, 3. Herein, we performed the gene expression profile analysis and identified the metabolic-phenotype differences between MEC1 and MC3 cells. Compared with MEC1 cells, MC3 cells acquired the mesenchymal-like characteristics and displayed the increased dependency on glutamine utilization. Mechanistic study indicates that the increased Akt activity and c-Myc stability play key roles in the induction of ASCT2-dependent glutaminolysis and the tumor aggressive phenotype, responding to the loss of NDRG2 expression in metastatic MC3 cells. Due to the predominant role of ASCT2 in driving glutaminolysis and tumor metastasis, we suppose targeting glutaminoysis represents an attractive strategy for the treatment of metastatic tumors.

## Results

### The aggressive derivatives of MEC cells exhibit the loss of epithelial phenotype

**Figure 1:**
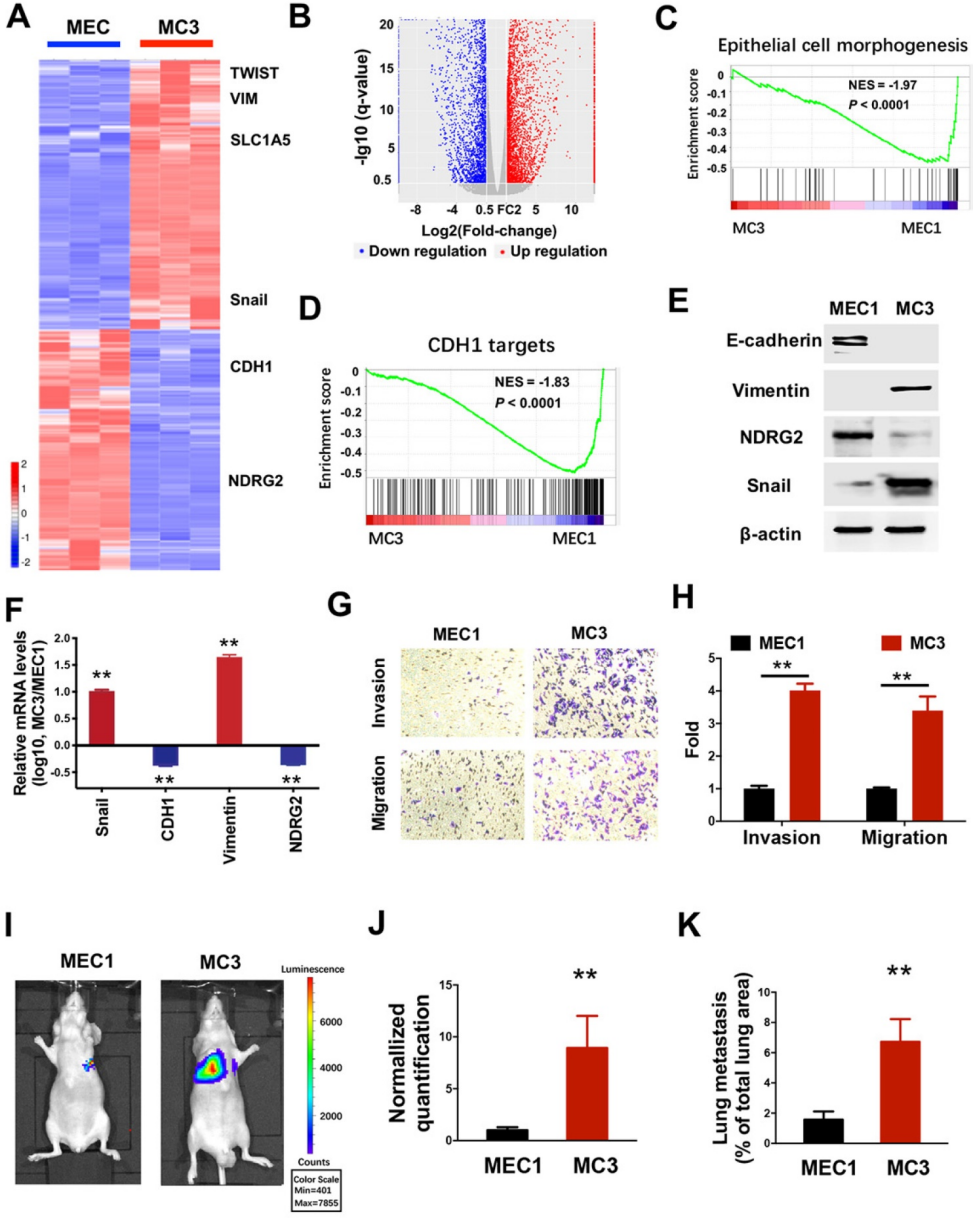
** The aggressive derivatives of MEC cells exhibit the loss of epithelial phenotype.** (A,B) Heatmap (A) and volcano plot (B) representing the genes significantly differentially expressed in MEC1 and MC3 cells (*P*<0.05). (C, D) GSEA analysis predicts the gene enrichment in epithelial cell morphogenesis (C) and CDH1 targets (D). (E, F) The indicated protein or mRNA levels were determined by western blotting (E) or qPCR assay (F) in MEC1 and MC3 cells. (G, H) The migratory and invasive behavior of MEC1 and MC3 cells were determined (G) and quantified (H). (I-J) Representative images (I) and statistical analysis (J) of the lung metastasis upon tail vein injection of MEC1 or MC3 cells. (K) Quantification of metastatic area in lungs (area of metastatic lesions, %of total lung area). Data are expressed as means ± SD (n = 3). **, *P* <0.001.

To gain mechanistic insight into system-level differences between primary tumors and their malignant derivatives, we determined the gene expression profiles in both MEC1 and metastatic MC3 cells (Figure 1A). Compared with the parental MEC1 cells, 4195 genes were downregulated and 4649 genes were up-regulated in MC3 cells (Figure 1A, 1B and Supplementary Table 1). Notably, gene set enrichment analysis (GSEA) revealed that the reduced enrichments of epithelial phenotype and E-cadherin (CDH1) targets were observed in MC3 cells (Figure 1C and 1D), suggesting the epithelial-mesenchymal transition (EMT) occurs in the metastatic MC3 cells. To verify this phenotype, we determined the EMT-related gene expressions. MC3 cells shows decreased epithelial marker E-cadherin and increased mesenchymal marker Vimentin expression, which might be attribute to the elevated Snail expression (Figure 1E and 1F). Accordingly, MC3 cells have higher invasion and migration abilities than MEC1 cells (Figure 1G and 1H). The tumor metastasis to the lungs was also increased in MC3 cells when cells were injected *via* the tail vein (Figure 1I-K). Thus, we demonstrate that the aggressive derivatives of MEC cells have higher metastatic ability, which might be attribute to the generation of EMT phenotype.

### Glutamine addiction occurs in mesenchymal MC3 cells

**Figure 2:**
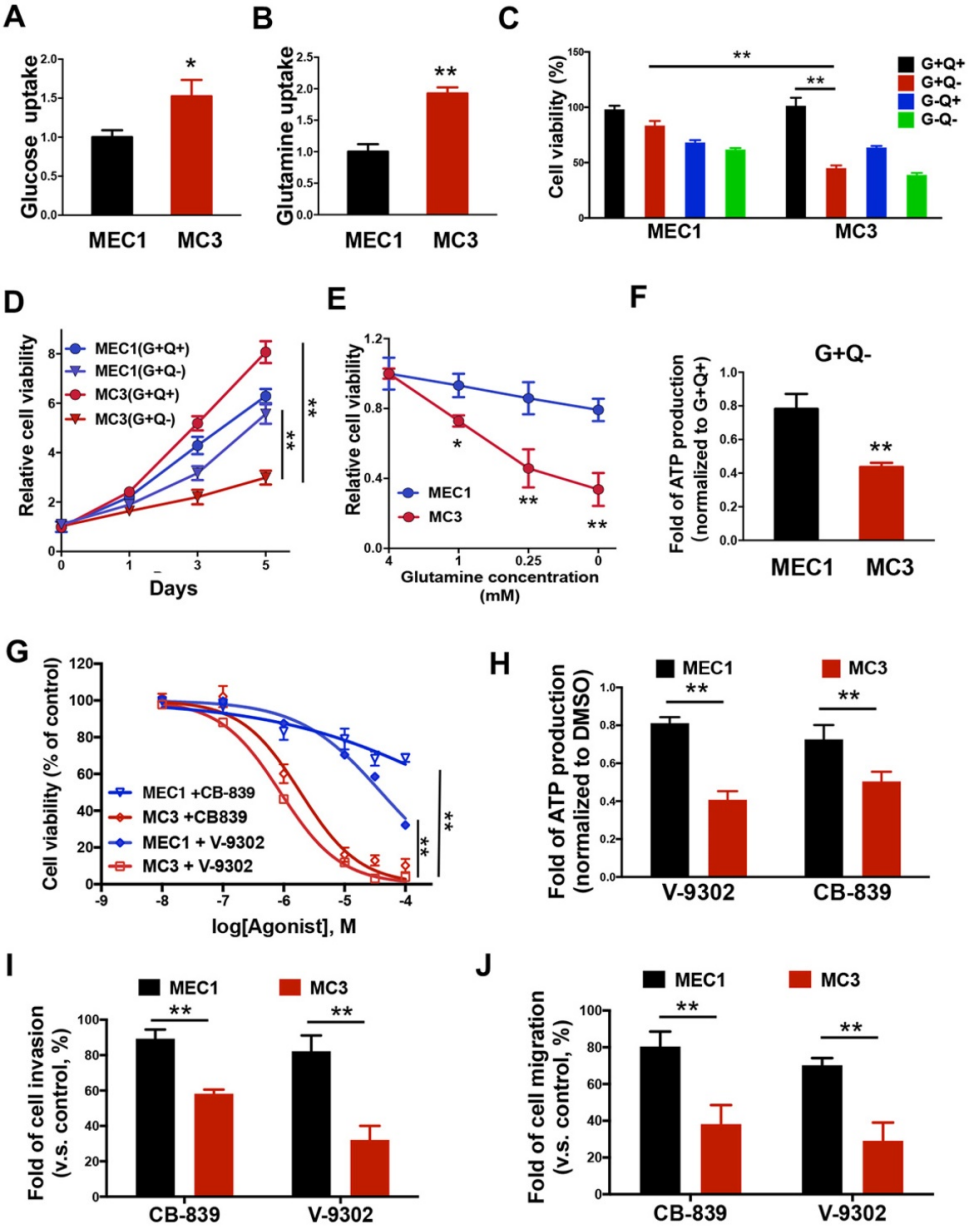
** Glutamine addiction occurs in mesenchymal MC3 cells.** (A, B) The glucose (A) or glutamine (B) uptake rate was determined in MEC1 and MC3 cells. (C) The normalized cell viability of MEC1 and MC3 cells grown in indicated conditions for 72 h. (D, E) Cells were grown with or without glutamine treatment for the indicated number of days (A) or with different dose of glutamine treatment for 72 h (E). Cell viability was normalized to its growth in complete medium containing glutamine. (F) The internal ATP levels were determined in medium lacking glutamine for 24 h and normalized to the levels in medium containing glutamine. (G) The cell viability was determined in MEC1 or MC3 cells following 0-100 µM CB-839 or V-9302 treatment for 72 h. (H) The internal ATP levels were determined in cells with 0.1 µM CB-839 or V-9302 treatment for 24 h, and normalized to the DMSO treatment group. (I, J) Quantification of the invasion (I) or migration (J) behavior of MEC1 and MC3 cells with or without 0.1 µM CB-839 or V-9302 treatment for 24 h. “G”, glucose; “Q”, glutamine. Data are expressed as means ± SD (n = 3). * *P* <0.01. **, *P* <0.001

Metabolic reprogramming is commonly observed in various cancers 35-37. Cancer cells show the increased glucose and glutamine metabolism to fuel their bioenergetic and biosynthetic demands. To investigate the differences of nutrient utilizations between primary and metastatic tumors, we determined how glucose, or glutamine, two important energy sources; are necessary for MEC cell survival. Intriguingly, MC3 cells can uptake more glucose and glutamine than MEC1 cells (Figure 2A and 2B). To better understand which nutrient is more important for cell survival, we tested the cells for growth in medium lacking glucose (G-Q+), glutamine (G+Q-), or both (G-Q-). Compared with MEC1 cells, metastatic MC3 cells are more sensitive to glutamine deprivation (Figure 2C-E), suggesting that there is a consistent variation in glutamine metabolism associated with tumor metastasis. Accordingly, the intracellular ATP levels were more dramatically reduced in MC3 cells than MEC1 cells after glutamine deprivation (Figure 2F).

To further confirm the glutamine dependency in metastatic MC3 cells, we blocked glutaminolysis through treating cells with glutaminase (GLS1) inhibitor CB-839 or glutamine metabolism inhibitor V-9302. Similar to the results in cells with glutamine deprivation, MC3 cells were more sensitive to either inhibitor treatment than MEC1 cells (Figure 2G), accompany with the reductions of ATP levels (Figure 2H). Moreover, the more dramatically reductions of cell invasion and migration abilities were observed in MC3 cells with low-dose of CB-839 or V-9302 treatment that does not influence cell proliferation (Figure 2I and 2J). Together, these findings suggest that mesenchymal cancer cells depend on glutamine-dependent pathways for survival, which contributes to cell growth and metastasis.

### NDRG2 suppresses MC3 cell survival through the blockage of glutaminolysis

**Figure 3:**
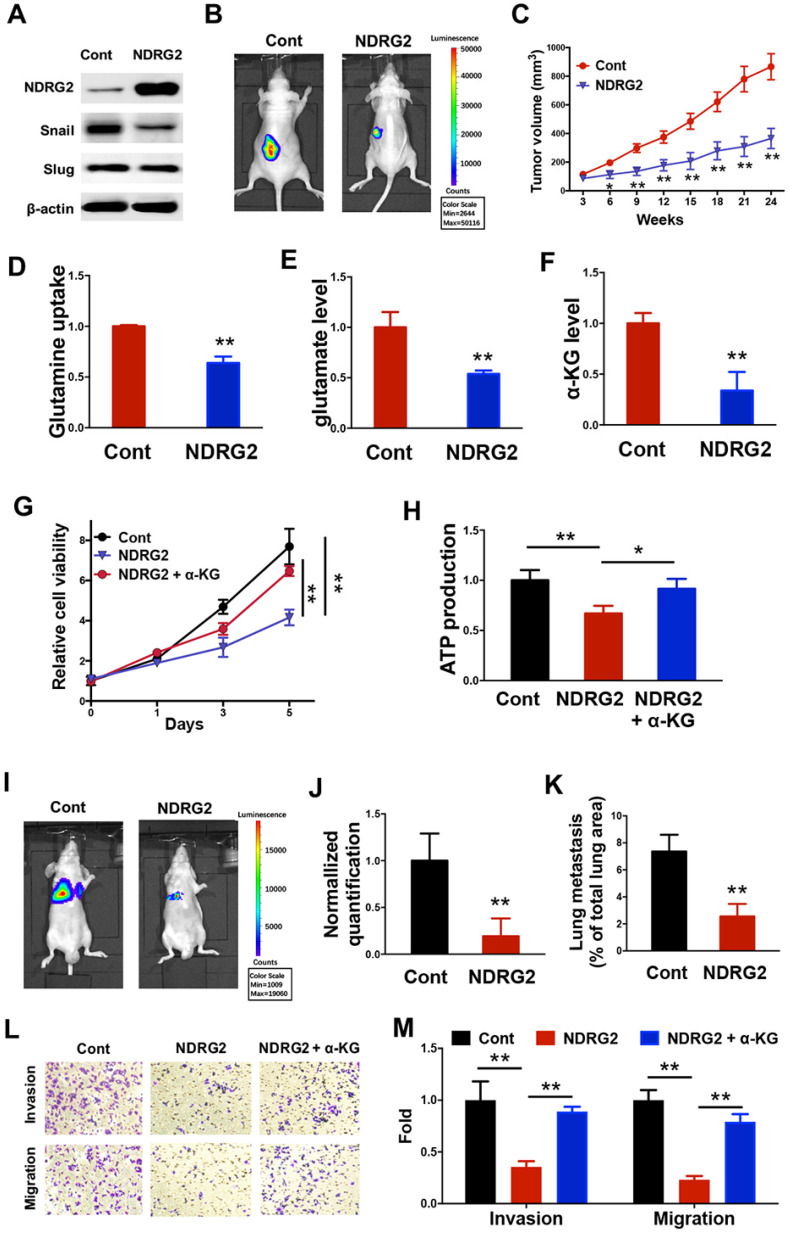
** NDRG2 suppresses MC3 cell survival through the blockage of glutaminolysis.** (A) Western blotting analysis of indicated protein expressions in MC3 cells with or without NDRG2 expression. (B) Representative bioluminescence imaging photographs of tumor burden 4 weeks after the subcutaneous injection of indicated cells. (C) The Tumor size was measured over a 4-week period and tumor volume was calculated by the formula (width^2^× length × 0.5). (D-F) The glutamine uptake rate (D), internal glutamate levels and α-KG levels were determined in MC3 cells with or without NDRG2 expression. (G, H) The cell viability (G) or internal ATP levels (H) were determined in indicated cells with or without α-KG treatment. (I-J) Representative images (I) and statistical analysis (J) of the lung metastasis upon tail vein injection of MC3 cells with or without NDRG2 expression. (K) Quantification of metastatic area in lungs (area of metastatic lesions, %of total lung area). (L, M) The migratory and invasive behavior of indicated cells were determined (G) and quantified (H). Data are expressed as means ± SD (n = 3). * *P* <0.01. ** *P* <0.001.

We further sought to determine the key modulators related to the loss of epithelial phenotype and the generation of glutamine-dependent phenotype in the metastatic tumor cells. Based on the gene expression profile data, we uncovered and verified that NDRG2 expression was lost in MC3 cells (Figure 1A, 1E and 1F). Due to the tumor-suppressive function of NDRG2 *via* regulating EMT and metabolic reprogramming in multiple types of cancers 38, 39, we suppose the generation of metastatic tumor specific phenotype might be associated with NDRG2 downregulation in MC3 cells. Thus we took advantage of lentivirus to gain of NDRG2 function through overexpression in MC3 cells (Figure 3A). NDRG2 protein level in MEC1 is comparable to that in NDRG2-overexpressed MC3 cells, which would rule out the possibility of experimental artifact (Supplementary Figure 1). NDRG2 expression dramatically diminished the growth rate of MC3 cells (Figure 3B and 3C). Since glutamine can enter the TCA cycle by being converted to glutamate and subsequently to α-ketoglutarate (α-KG), we observed that NDRG2 expression decreased the intracellular glutamine, glutamate and α-KG levels (Figure 3D-F), suggesting the involvement of NDRG2-repression of glutaminolysis in cell growth inhibition. To further confirm this hypothesis, we treated MC3 cells with α-ketoglutarate (α-KG), and found that the reduced cell growth rate and intracellular ATP levels by NDRG2 were partly resumed after α-KG treatment (Figure 3G and 3H).

Snail and Slug are the key transcription factors in regulating EMT. In addition to regulating cell survival, NDRG2 overexpression also repressed the expression of Snail (Figure 3A), indicating NDRG2 probably plays a role in regulating EMT and metastasis in MC3 cells. Consistent with this hypothesis, NDRG2 overexpression decreased MC3 cell invasion and migration abilities, and suppressed metastatic tumor formation in lung of nude mice (Figure 3I-M). Whereas the α-KG treatment restored cell invasion and migration (Figure 3L and 3M), suggesting NDRG2 restricts cell metastasis as a metabolic checkpoint in MEC cells. Thus, we demonstrate that the generations of EMT and glutamine-dependent phenotypes are attributed to the loss of NDRG2 expression in metastatic tumors, and the gain of NDRG2 function can suppress metastatic tumor survival through the blockage of glutaminolysis.

### NDRG2 represses ASCT2 transcription

**Figure 4:**
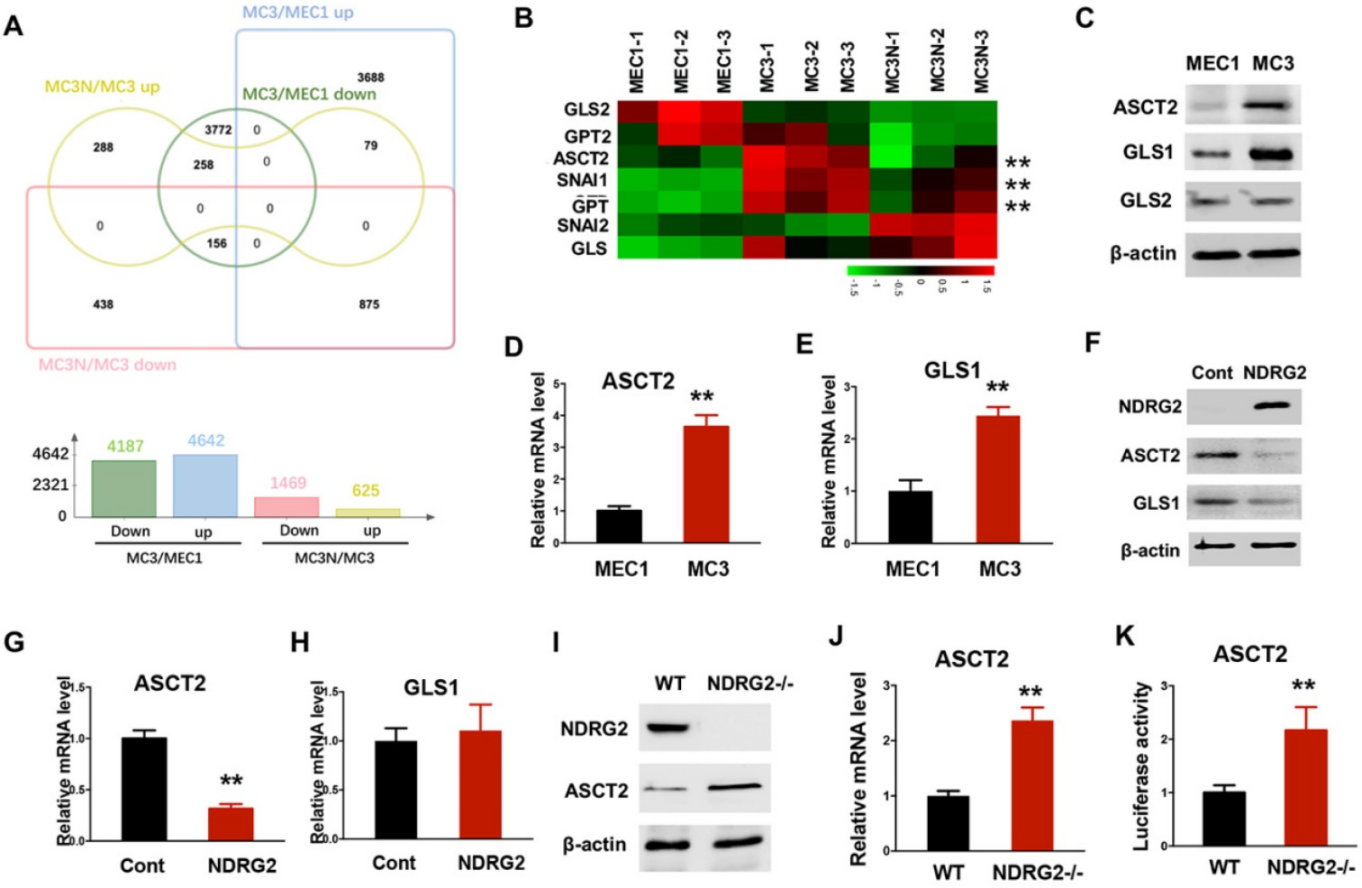
** NDRG2 represses ASCT2 transcription.** (A) The differentially expressed genes were clustered and calculated in MEC cells, MC3 cells, and MC3 cells with NDRG2 expression (MC3N). Venn diagram was made with Jvenn (http://jvenn.toulouse.inra.fr/app/index.html). (B) The heatmap represents the expression of glutaminolytic-related genes in indicated cells. (C-E) The indicated protein levels (C), and ASCT2 (D) and GLS1 (E) mRNA levels were determined by western blotting and qPCR, respectively in MEC1 and MC3 cells. (F-H) The indicated protein levels (F), and ASCT2 (G) and GLS1 (H) mRNA levels were determined by western blotting and qPCR, respectively in MC3 cells with or without NDRG2 overexpression. (I-K) The indicated protein levels (I), ASCT2 mRNA levels (J) and ASCT2 promoter activity (K) were determined respectively. Data are expressed as means ± SD (n = 3). **, *P* <0.001.

To fully address the differences of glutamine metabolism between primary and metastatic tumors related to NDRG2, we carried out RNA-seq assay to determine the gene expression profile alterations in MC3 cells with or without NDRG2 overexpression. A total of 2094 gene expressions were markedly changed after NDRG2 overexpression (Figure 4A and Supplementary Table 2). Compared with MEC1 cells, we identified 875 genes increased in MC3 cells whereas decreased after NDRG2 overexpression, and 258 genes decreased in MC3 cells whereas increased after NDRG2 overexpression (Figure 4A, Supplementary Table 1-2). The expressions of the key enzymes involved in glutaminolysis (ASCT2, GLS, GPT) were increased in MC3 cells (Figure 4B). Intriguingly, ASCT2 and GPT levels were markedly decreased after NDRG2 overexpression (Figure 4B). Since ASCT2 as a major glutamine transporter were found highly expressed in numerous cancers 31, 40, we thus would like to further confirm the reciprocal relationship between NDRG2 and ASCT2. Consistent with high rate of glutaminolysis, MC3 cells expresses high levels of ASCT2 and GLS1 than MEC1 cells (Figure 4C-E). Whereas, NDRG2 expression leads to a dramatic reduction of ASCT2 in both protein and mRNA levels (Figure 4F-4G). NDRG2-dependent suppression of GLS1 were observed in protein levels but not in mRNA levels (Figure 4F and 4H), indicating NDRG2 may also regulate GLS1 in post-transcriptional level. Further, we introduced NDRG2-null murine embryonic fibroblasts (MEFs) to determine the effect of NDRG2 on ASCT2 expression. Accordingly, the increased ASCT2 expression and ASCT2 luciferase reporter activity were observed in NDRG2-knockout MEFs (Figure 4I-K). These data suggest that NDRG2-dependent repression of glutaminolysis suppresses the aggressive phenotype of metastatic tumors potentially through the repression of ASCT2 transcription.

### NDRG2 represses ASCT2 through the induction of Fbw7-dependent c-Myc degradation

**Figure 5:**
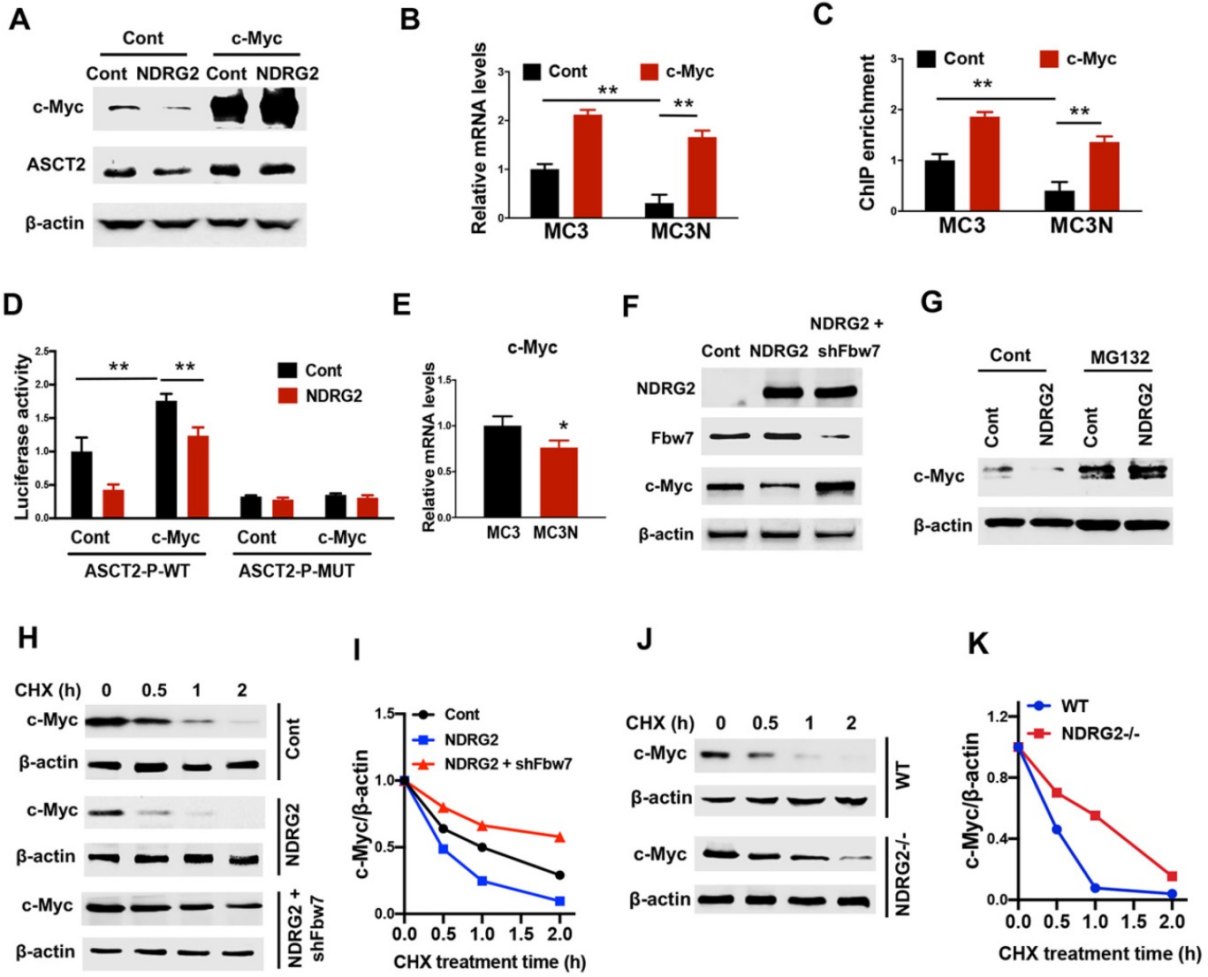
** NDRG2 represses ASCT2 through the induction of Fbw7-dependent c-Myc degradation.** (A-D) We gained c-Myc function through overexpression in NDRG2-overexpressing MC3 cells. The indicated proteins levels (A), ASCT2 mRNA levels (B), c-Myc enrichment on ASCT2 promoter (C), and the activities of wild-type and E-box-mutant ASCT2 promoter (D) were determined respectively. (E) c-Myc mRNA levels were determined in MC3 cells with or without NDRG2 expression. (F) Levels of indicated protein expressions were determined in MC3 cells with or without NDRG2 expression, or NDRG2-overexpressed MC3 cells with Fbw7 knockdown. (G) MC3 cells with or without NDRG2 expression were treated with 20 µM MG132 for 2 h. Levels of indicated proteins were determined by western blot. (H-K) Levels of indicated proteins in indicated MC3 cells or MEF cells following 10 µg/ml CHX treatment for indicated time (H and J). c-Myc expression levels were further quantified by ImageJ and normalized to β-actin to determine the protein degradation rate (I and K). **, *P* <0.001.

Since accumulated evidence show that c-Myc regulates a transcriptional program to stimulate glutaminolysis, including ASCT2 17, we suppose NDRG2 represses ASCT2 potentially through c-Myc suppression. Expectedly, ectopic expression of NDRG2 leads to a remarkable reduction of c-Myc (Figure 5A). The gain of c-Myc function restored ASCT2 protein and mRNA levels which were repressed by NDRG2 (Figure 5A and 5B). Further, using JASPAR program (http://jaspar.genereg.net) 41, we identified an E-box cis-elements on ASCT2 promoter. NDRG2 overexpression significantly decreased c-Myc occupancy on ASCT2 promoter (Figure 5C). Whereas c-Myc overexpression blocked NDRG2 suppressive effect on c-Myc enrichment and ASCT2 promoter activity (Figure 5C and 5D), but failed to activate ASCT2 promoter after the mutation of E-box element (Figure 5D). Thus, NDRG2 appears to block c-Myc binding to ASCT2 promoter elements and this binding is associated with ASCT2 expression levels.

Though c-Myc mRNA expression decreased after NDRG2 overexpression (Figure 5E), the dramatically reduction of c-Myc protein levels potentially not be attribute to the minimal variation of c-Myc mRNA abundance in NDRG2-overexpressed MC3 cells, raising the possibility that NDRG2 regulates c-Myc in post-translational level. As expected, c-Myc stability was attenuated in NDRG2-overexpressed MC3 cells and enhanced in NDRG2-knockout MEF cells following cycloheximide (CHX) treatment (Figure 5F-K). Fbw7 (F-box and WD repeat domain-containing 7) is an E3 ubiquitin ligase and targets many substrates for proteasomal degradation, including c-Myc 42. Knockdown Fbw7 restored c-Myc protein expression and stability which were repressed by NDRG2 (Figure 5F, 5H-K). Thus, NDRG2 represses c-Myc potentially through the induction of Fbw7-dependent c-Myc degradation, which confers to the suppression of c-Myc transcriptional regulation of ASCT2.

### NDRG2 suppression of c-Myc stabilization is mediated by AKT/GSK3β pathway

**Figure 6:**
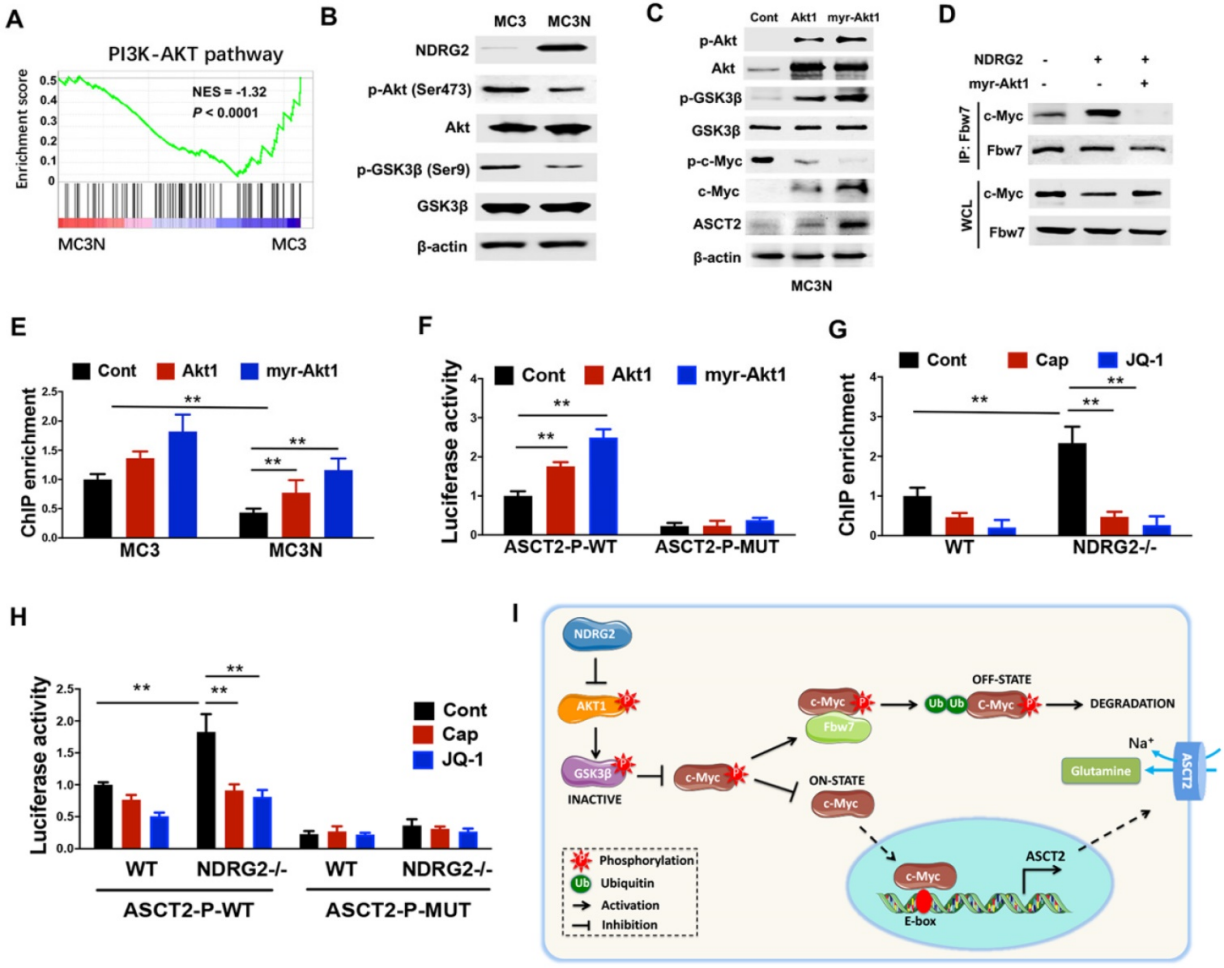
** NDRG2 suppression of c-Myc stabilization is mediated by AKT/GSK3β pathway.** (A) GSEA analysis predicts the gene enrichment in PI3K-Akt pathway in MC3 cells with or without NDRG2 expression. (B) Levels of indicated proteins in MC3 cells with or without NDRG2 expression. (C-F) We overexpressed the wild type or activating form of Akt1 (myr-Akt1) in NDRG2-overexpressing MC3 cells. (C) The indicated proteins levels were determined by western blotting. (D) Immunoprecipitation was performed to determine the interaction between c-Myc and Fbw7 in indicated MC3 cells. After the Fbw7 protein were immunoprecipitated with an anti-Fbw7 antibody, indicated proteins were detected by western blotting. (E-F) c-Myc enrichment on ASCT2 promoter (E) and the activities of wild-type and E-box-mutant ASCT2 promoter (F) were determined respectively. (G, H) c-Myc enrichment on ASCT2 promoter (G), and the activities of wild-type and E-box-mutant ASCT2 promoter (H) were determined in wild-type or NDRG2-/- MEF cells, respectively. Data are expressed as means ± SD (n = 3). (I) The proposed mechanism of NDRG2 action in regulation of Akt/c-Myc axis and ASCT2 expression. **, *P* <0.001.

GSK3β facilitates c-Myc turnover through promoting c-Myc (Thr58) phosphorylation, and subsequently promotes c-Myc for ubiquitination and degradation by Fbw7 42. Whereas Akt activation stabilizes c-Myc through phosphorylating GSK3β Ser9, which is associated with GSK3β inactivation 43, raising the possibility that NDRG2 suppression of c-Myc stabilization is mediated by AKT/GSK3β pathway. Notably, GSEA analysis shows that the enrichment of Akt pathway in NDRG2-overexpressed MC3 cells was dramatically reduced (Figure 6A). The decreased Akt (Ser473) and GSK (Ser 9) phosphorylation were further confirmed in NDRG2-overexpressed MC3 cells (Figure 6B). Thus we further recovered Akt activity by the ectopic expression of either Akt1 or a constitutively active Akt1 that contained a myristoylation sequence (myr-Akt1). The remarkable increase of c-Myc expression and decrease of c-Myc (T58) phosphorylation were observed after Akt1 or myr-Akt1 overexpression in NDRG2-overexpressed MC3 cells (Figure 6C). Especially, NDRG2 expression increased the interaction of c-Myc with Fbw7, which was blocked by Akt activation (Figure 6D). Therefore, c-Myc downregulation is through the activation of GSK-3β activity which is mediated by the suppression of Akt activity in response to NDRG2 expression.

Further, we determined whether NDRG2 suppression of ASCT2 is through repressing Akt/c-Myc axis. Akt1 overexpression increased c-Myc occupancy on ASCT2 promoter, and increased ASCT2 promoter activity which was suppressed after the c-Myc-binding E-box mutation (Figure 6E and 6F). Consistent with above data, the enrichment of c-Myc on ASCT2 promoter was also increased in NDRG2-/- MEF cells (Figure 6G). Whereas the inductions of c-Myc enrichment and ASCT2 promoter activity were blocked by either Akt inhibitor Capivasertib or c-Myc inhibitor JQ1 (Figure 6G and 6H). Therefore, we here demonstrate that NDRG2 suppresses c-Myc expression *via* the inhibition of Akt and GSK3β activation which is associated with Fbw7-depdent c-Myc degradation, and consequently blocks c-Myc transcriptional activity on ASCT2 promoter (Figure 6I).

### NDRG2 repression of ASCT2 controls EMT progression and cell survival in metastatic tumor

**Figure 7:**
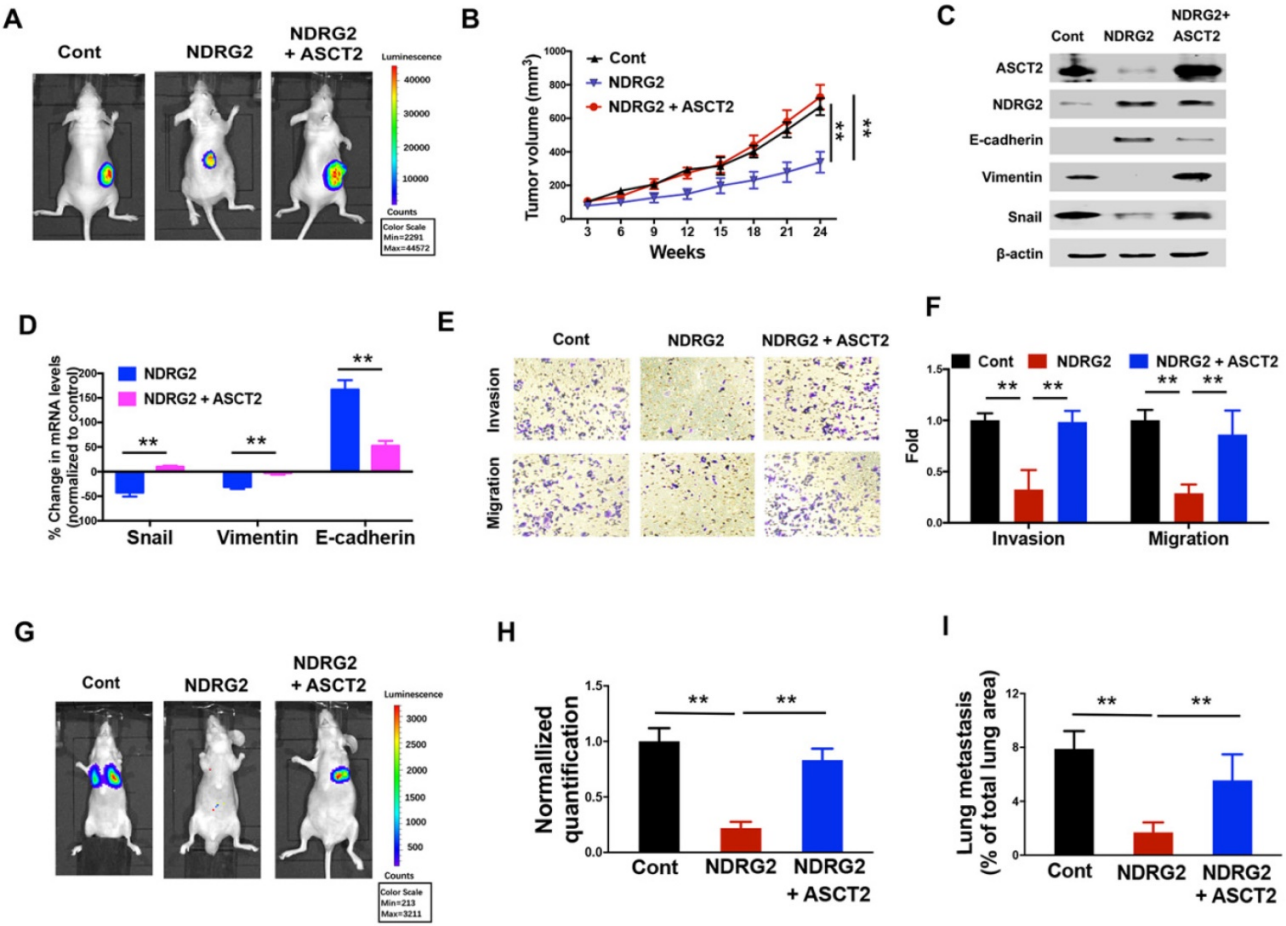
** NDRG2 repression of ASCT2 controls EMT progression and cell survival in metastatic tumor.** We gained ASCT2 expression in NDRG2-ovexpressing MC3 cells and determined the cell biological behaviors. (A) Representative bioluminescence imaging photographs of tumor burden 4 weeks after the subcutaneous injection of indicated cells. (B) The Tumor size was measured and tumor volume was calculated. (C, D) Levels of indicated protein (C) or mRNA (D) expressions were determined by western blotting or qPCR respectively. (E, F) The migratory and invasive behavior of indicated cells were determined (E) and quantified (F). (G, H) Representative images (G) and statistical analysis (H) of the lung metastasis upon tail vein injection of indicated cells. (I) Quantification of metastatic area in lungs (area of metastatic lesions, %of total lung area). Data are expressed as means ± SD (n = 3). *, *P* <0.01. **, *P* <0.001,

Given the crucial function of ASCT2 for glutamine utilization and energy production in numerous cancers, we then sought to determine whether the dramatic alteration of ASCT2 levels in response to NDRG2 is functionally linked to NDRG2 suppression of metastatic tumor cell survival and the aggressive phenotype generation. The overexpression of ASCT2 restored the growth inhibitory effect of NDRG2 on MC3 cells (Figure 7A and 7B). Further, ASCT2 overexpression acquired the mesenchymal expression profile which was repressed NDRG2, as evidenced by the increased accumulations of Snail and Vimentin, and decreased accumulation of E-cadherin (Figure 7C and 7D). In accordance with mesenchymal phenotype, ASCT2 overexpression increased cell invasion and migration abilities in NDRG2-overexpressed MC3 cells (Figure 7E and 7F). The establishment of metastatic foci in lung was also promoted by ASCT2 in nude mice (Figure 7G-I). Thus, we conclude that suppression of ASCT2 is required for NDRG2-dependent inhibition of cell growth, EMT and metastasis in metastatic tumor cells.

### The interplay between NDRG2 and ASCT2 dictates tumor malignancy

**Figure 8:**
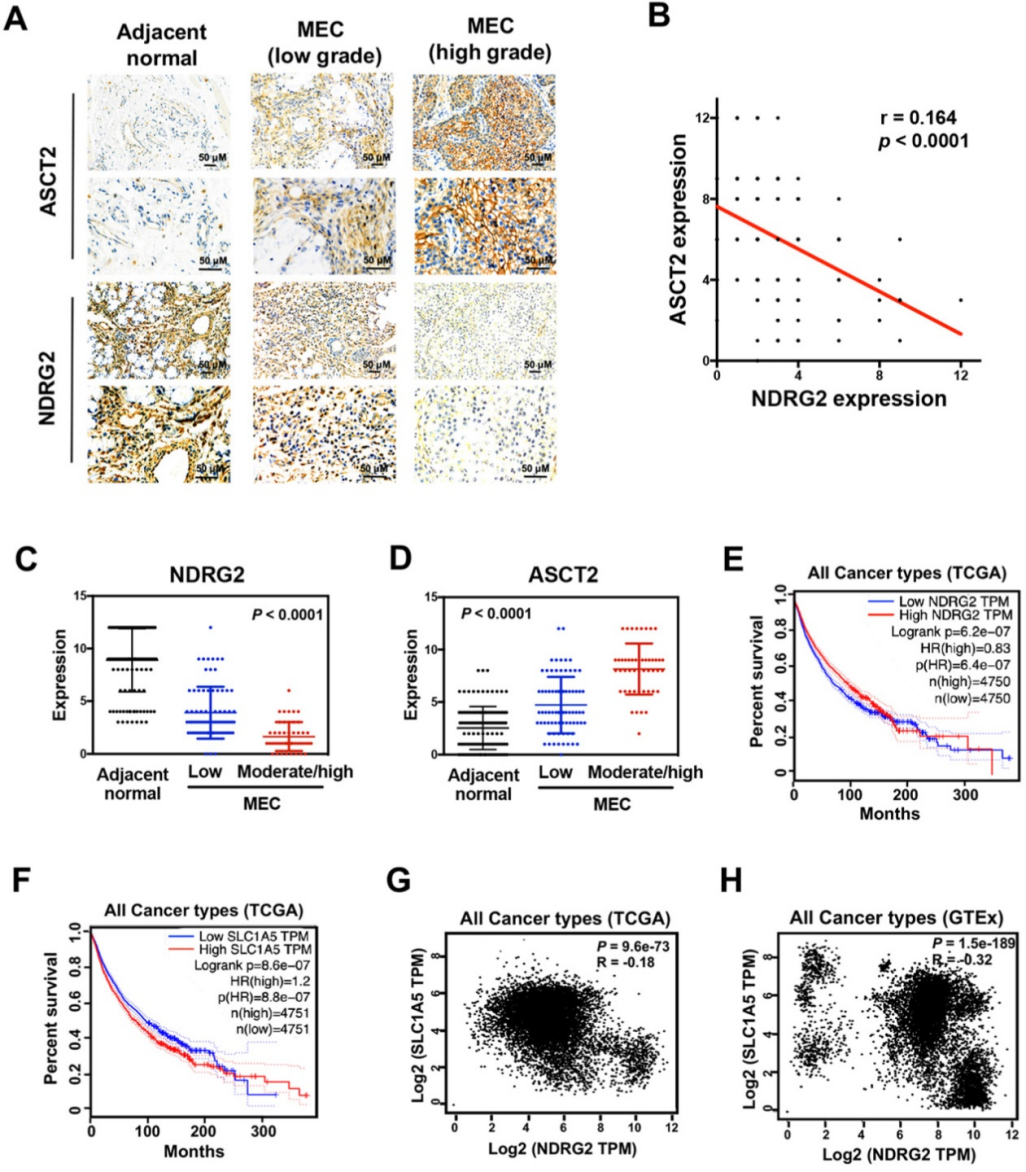
** The interplay between NDRG2 and ASCT2 dictates tumor malignancy.** (A) Immunohistochemistry staining of NDRG2 and ASCT2 in human adjacent normal and MEC tissues. (B) Negative correlation between NDRG2 and ASCT2 expressions with linear regression and Pearson's correlation significance (*P* <0.0001, ANOVA test). (C, D) NDRG2 (C) or ASCT2 (D) expression by immunohistochemistry staining in MEC tissues at different grade. (E-F) Survival analysis of NDRG2 (E) or ASCT2 (F) association with prognosis in all cancer types by GEPIA. (G-H) Negative correlation between NDRG2 and ASCT2 mRNA expression patterns in all cancer types from TCGA (G) or GTEx (H) data set by GEPIA with linear regression and Pearson's correlation significance (*P* <0.001, ANOVA test).

To measure whether there is a clinical correlation between NDRG2 and ASCT2 in human tissue samples, and whether these proteins are correlated with MEC progression, we performed immunohistochemical analysis to assess the expression levels of NDRG2 and ASCT2 in 125 cases of MEC patients. NDRG2 was broadly lowly expressed in MEC tissues compared with adjacent normal tissues, whereas the expression pattern of ASCT2 was the inverse association of NDRG2 (Figure 8A-78). Especially, the high-grade MEC tissues, which have the high metastatic potentials 3, exhibit a more remarkable expression pattern of high ASCT2 and low NDRG2 (Figure 8A, 8C and 8D). Therefore, the expression levels of NDRG2 and ASCT2 were negatively and positively associated with MEC malignancy respectively. The reciprocal relationship between NDRG2 and ASCT2 plays a key role in the regulation of MEC aggressive phenotype. To verify the NDRG2 regulation of ASCT2 generally occurs in various cancers, we performed the *in silico* analysis in different types of tumor tissues of the multidimensional data set from TCGA (the cancer genome atlas) and GTEx (Genotype-Tissue Expression). Survival analysis revealed that either the low NDRG2 expression or high ASCT2 expression can predict the poor outcome of patients (Figure 8E and 8F). Further, the significant negative correlation of NDRG2 and ASCT2 mRNA expression levels was observed in the tissues of all cancers in both TCGA and GTEx datasets (Figure 8G and 8H), confirming our proposal that NDRG2 suppresses ASCT2 expression in transcriptional level.

## Discussion

Glutamine provides nitrogen for protein and nucleotide biosynthesis in growing cells. However, in glutamine-dependent cancer cells, the high rate of glutamine uptake does not appear to result solely from its role as a nitrogen donor 11. The increased glycolysis lead to the ''empties'' of the TCA cycle in mitochondria. Glutamine ''refills'' the TCA cycle by a process termed anapleurosis, which is critical for maintaining the mitochondrial function for metabolism 44, 45. Our study demonstrated a glutamine-dependent phenotype in the metastatic MC3 cells. Compared with primary MEC1 cells, MC3 uptakes more glutamine for survival, whereas the blockage of glutaminolysis, by either glutamine withdrawal or glutaminolysis inhibitors, resulted in a more dramatic decrease of MC3 cell survival. Based on the malignant traits of MC3 cells associated with the EMT program execution (Figure 1A-F), we demonstrate that metabolic pathway related to glutamine is not only critical for cell proliferation, but also specifically enable carcinoma cells to acquire mesenchymal-like characteristics, and further promote metastatic dissemination from primary tumors.

Mesenchymal cells acquire the characters associated with high-grade malignancy, such as dedifferentiation, invasiveness, and the resistance to apoptosis and chemotherapy, which results in the metastatic dissemination from primary tumors 21 Thus, inhibiting the EMT may maintain the lower-grade state of epithelial-derived cancer cells, potentially suppressing metastasis and increasing therapeutic efficacy. Confirming this point, studies showed that suppression of transcription factors, such as Snail or ZEB1, results in the inhibition of EMT and induction of chemoimmunosensitization in metastatic cancer cells 46-48. However, the development of inhibitors targeting EMT-related transcription factors remains a challenge 49. Here, we demonstrated that mesenchymal cells increase the glutamine utilization and exhibit glutamine-dependent phenotype, potentially due to the increased expressions of ASCT2 and GLS1, suggesting that the EMT program probably be modulated through inhibition of glutaminolytic enzymes. The feasibility is reinforced in this study showing that the growth and invasiveness of mesenchymal MC3 cells are more sensitive to ASCT2 or GLS1 inhibitor treatment (Figure 2G-J). Thus, targeting metabolic enzyme or the key modulators related to glutaminolysis may provide new strategy for the treatment of metastatic tumors.

We and other groups observed the inhibitory effect of NDRG2 on EMT progression, potentially through the inhibition of STAT3 pathway or activation of GSK3β activity 38, 50. Consistent with these findings, we identified the lost expression of NDRG2 in mesenchymal MC3 cells. NDRG2 expression can not only ablate EMT program but also suppress cell survival, accompany with the decreased glutaminolytic rate. Thus, the generation of glutamine-dependent phenotype in metastatic tumor is probably attributable to NDRG2 downregulation. Although glutamine has many intracellular fates, a cell permeable analog of a tricarboxylic acid cycle (TCA) intermediate, α-KG, blocked NDRG2 suppressing both cell growth and metastasis, indicating the pleiotropic role of NDRG2 in metastatic tumors is dependent on the blockage of glutaminolysis pathway.

Numerous studies demonstrated that ASCT2 mediates the entry of glutamine to the glutaminolysis pathway to generate building blocks and energy for anabolic purposes 13, 14, 51, which is responsible for the activation of mTORC1-dependent signaling 13. Here, we found that ASCT2 is upregulated in mesenchymal MC3 cells and repressed by NDRG2 overexpression. The gain of ASCT2 function in NDRG2 overexpressing cells restored the mesenchymal phenotype in MC3 cells potentially through blocking NDRG2 suppression of Snail. Due to the crucial role of mTORC1 in regulation of EMT and Snail expression 52, 53, we suppose that ASCT2 modulates Snail expression through the glutamine-dependent activation of mTORC1 pathway. Nonetheless, further studies are needed to fully address the association of glutamine-dependent anapleurosis with EMT progression in response to glutamine transporter or glutaminase activation.

Subjective evidence demonstrated that c-Myc stimulates glutaminolysis by activating, directly or indirectly, the expression of genes involved in glutamine uptake and utilization, including ASCT2 17, 54. Our experiments extend these findings by demonstrating suppression of c-Myc is essential for NDRG2-dependent inhibition of ASCT2 expression and glutaminolysis (Figure 6I). Especially, we identified the c-Myc specific binding E-box element on ASCT2 promoter, the mutation of which can abolish c-Myc enrichment on ASCT2 promoter and ASCT2 luciferase activity. Based on our previous findings that the diminished NDRG2 expression in cancer cells is predominantly due to transcriptional repression of its promoter by high levels of c-Myc 38, we suggest that NDRG2 and c-Myc can form a feedback loop, which is involved in the rewiring of glutamine metabolism.

It is widely accepted that elevated c-Myc expression is dependent on the aberrant activation of PI3K/Akt signaling, which can repress GSK3β-mediated c-Myc (T58) phosphorylation and Fbw7-dependent c-Myc degradation 42, 43. Based on GESA analysis, we observed the reduced enrichment of Akt pathway after NDRG2 overexpression. As NDRG2 has been shown represses PI3K/Akt activity via the dephosphorylation of PTEN 55, it is reasonable to assume that NDRG2 suppression of c-Myc transcriptional activity is through the blockage of Akt pathway. Confirming this point, NDRG2 expression dramatically reduced the phosphorylated Akt and GSK3β levels, and increased c-Myc phosphorylation and Fbw7-dependent degradation. Accordingly, the constitute Akt activation in NDRG2 overexpressing MC3 cells results in the marked decrease of Fbw7/c-Myc interaction and increase of c-Myc enrichment on ASCT2 promoter, which was ablated by either Akt inhibitor Capivasertib or c-Myc inhibitor JQ1. Thus, c-Myc induction of ASCT2 is dependent on Akt activation in response to the loss of NDRG2 in metastatic tumors (Figure 6I). Although the tendency of Myc to complement PI3K/Akt is related to the interdependence of glutamine and glucose metabolism in support of cell growth remains controversial 17, 56, 57, we suggest that NDRG2 dependent suppression of c-Myc through PI3K/Akt pathway was at least one of the routes in terms of suppression of ASCT2 and glutaminolysis.

In summary, our data provide the first evidence that the mesenchymal phenotype generation in metastatic tumor cells is potentially due to the activation of glutaminolytic pathway. NDRG2 suppresses Akt signaling and promotes c-Myc degradation, which results in the ablation of ASCT2 expression and glutaminolysis (Figure 6I). As such, we point out that targeting glutaminolytic pathway may provide a new strategy for the treatment of metastatic tumors.

## Materials and Methods

### Cell culture and conditions

The human mucoepidermoid carcinoma cell line MEC1 and MC3 were established as previously described 3, and cultured in RPMI1640 (Hyclone) with 10% FBS 3. Nutrient depletion studies were performed using reconstituted medium without glucose and glutamine. Glucose or glutamine was added into the media to the final concentrations, 25 and 2 mM, respectively.

### Plasmid, virus and reagent

The pcDNA3.1-Akt1, pcDNA3.1-myr-Akt1, and pcDNA3.1-c-Myc were generated previously 58. The human ASCT2 promoter (-880 ~ +22) was subcloned into pGL4 vector. Lentivirus NDRG2 and ASCT2 were packaged in Hanbio Biotechnology (China). CB-839 (HY-12248) and V-9302 (HY-112683) were purchased from MedChemExpress. 2-NBDG (N13195) was from Invitrogen.

### Immunoprecipitation and Western blotting

Cells were harvest and extracted. The extracts were incubated with anti-Fbw7 antibody overnight at 4 °C. After blended with Protein A/G Dynabeads® (Thermofisher), the complex was washed twice with PBS, in turn to be resuspended and boiled with 2 X SDS loading buffer and subjected to Western blot analysis. Western blotting assay was performed as previously described 58. Primary antibodies were used at dilutions of 1:200 for anti-FBW7 (sc-293423, Santa Cruz), 1:1000 for anti-c-Myc (#ab32072, Abcam), anti-p-Akt (S473) (#4051, Cell Signaling Technology), anti-Akt (#2938, Cell Signaling Technology), anti-E-cadherin (#144725, Cell Signaling Technology), anti-Vimentin (#5741, Cell Signaling Technology), anti-NDRG2 (H00057447-M03, ABNOVA), anti-ASCT2 (#ab237704, Abcam), anti-Snail (#3879, Cell Signaling Technology), anti-Slug (#9585, Cell Signaling Technology), anti-GLS1 (#ab200408, Abcam), anti-GLS2 (#ab150474, Abcam) and anti-β-actin (#A5441, Sigma).

### Protein degradation assay

MC3 cells or MEF cells were incubated with CHX (10 μg/ml, Sigma) for indicated time. Cells were then harvest and western blotting was performed as described above.

### Quantitative PCR

The RNA was extracted and cDNA was generated through GoScript Reverse Transcription System (Promega). The quantitative PCR (qPCR) assay was performed as described previously 20. Primer sequences are available on request.

### Cell-based assays

MTT assay, glycolysis and glutamine relevant assays were performed as described previously 37-39, 59. The ATP production of cells was normalized to the total protein levels in each group, respectively.

### *In vitro* migration and invasion assays

Cell migration and invasion were performed by polycarbonate transwell filters containing 8 mm pores (Becton Dickinson Labware) with or without Matrigel (BD Biosciences) coated, as described previously 38. The migration and invasion indices were calculated as the mean number of cells in 10 random fields at ×20 magnification.

### ASCT2 promoter related assays

Luciferase reporter assay and Chromatin immunoprecipitation were performed as described previously 58. The sequences of PCR primers detecting ASCT2 promoter are available on request.

### RNA-seq experiments

The RNA were extracted from MEC1 cells, MC3 cells and MC3 cells with NDRG2 overexpression (n=3). Paired-end libraries were synthesized by using the TruSeq™ RNA Sample Preparation Kit (Illumina, USA) following TruSeq™ RNA Sample Preparation Guide. Briefly, the poly-A containing mRNA molecules were purified using poly-T oligo-attached magnetic beads. Following purification, the mRNA is fragmented into small pieces using divalent cations under 94 for 8 min. The cleaved RNA fragments are copied into first strand cDNA using reverse transcriptase and random primers. This is followed by second strand cDNA synthesis using DNA Polymerase I and RNase H. These cDNA fragments then go through an end repair process, the addition of a single 'A' base, and then ligation of the adapters. The products are then purified and enriched with PCR to create the final cDNA library. Purified libraries were quantified by Qubit® 2.0 Fluorometer (Life Technologies, USA) and validated by Agilent 2100 bioanalyzer (Agilent Technologies, USA) to confirm the insert size and calculate the mole concentration. Cluster was generated by cBot with the library diluted to 10 pM and then were sequenced on the Illumina NovaSeq 6000 (Illumina, USA).

### Mouse Models

The *in vivo* tumorigenicity assay was performed with MEC cells as previously described 33. For in tumor metastatic assay in mice, MEC cells with D-Luciferin (Promega, Madison, WI, USA) were injected intravenously via tail vein in nude mice to obtain lung tumors. The luminescence signal was recorded by using the Xenogen-IVIS Imaging System.

### Tissue samples and immunohistochemistry staining

This study was approved by the ethics committee of the Fourth Military Medical University. A total of 125 MEC samples were collected. The tumor diagnostic information and immunohistochemistry staining were performed as described previously 60. Student's t-test was applied for statistical analyses of the relative protein levels. The correlation between NDRG2 and ASCT2 expressions were analyzed with linear regression and Pearson's correlation significance.

### Patient data analysis

The Gene Expression Profiling Interactive Analysis (GEPIA) was used to determine the correlation of NDRG2 and ASCT2 in the dataset of different types of tumors. NDRG2 and ASCT2 were entered as the query genes and co-expression was provided through the GEPIA user interface. The survival and gene expression correlation analysis were obtained from TCGA and GTEx datasets respectively.

### Statistical Analysis

Data are expressed as mean±SD. Statistical analysis was performed with the SPSS10.0 software package by using student's *t-*test for independent groups. Statistical significance was based on a value of *P*≤0.05.

## Supplementary Material

Supplementary figures and tables.

## Abbreviations

EMT: epithelial-mesenchymal transition
ASCT2: alanine-serine-cysteine transporter 2
α-KG: α-ketoglutarate
MEC: mucoepidermoid carcinoma
NDRG2: N-Myc downstream regulated gene 2
Fbw7: F-box and WD repeat domain-containing 7
qPCR: quantitative PCR
GSEA: gene set enrichment analysis
TCA: tricarboxylic acid cycle
WT: wild type
