# Supplementary material for: NDRG2 ablation reprograms metastatic cancer cells towards glutamine dependence via the induction of ASCT2
Source: Int J Biol Sci. 2020 Oct 16;16(16):3100–15. doi: 10.7150/ijbs.48066 (PMC7645990; doi:10.7150/ijbs.48066)
Supplement: Supplementary file 1 — Supplementary figures and tables. [file ijbsv16p3100s1.zip › Supplementary figure legends.docx]

**Supplementary figure legends**

Figure 1 (A-C) NDRG2 mRNA (A) and protein (B) levels were determined in MEC1 cells , and MC3 cells with or without NDRG2 overexpression. The indicated protein levels were quantified by Image J. Data are expressed as means ± SD (n = 3).
