# Supplementary material for: NDRG2 ablation reprograms metastatic cancer cells towards glutamine dependence via the induction of ASCT2
Source: Int J Biol Sci. 2020 Oct 16;16(16):3100–15. doi: 10.7150/ijbs.48066 (PMC7645990; doi:10.7150/ijbs.48066)
Supplement: Supplementary file 1 — Supplementary figures and tables. [file ijbsv16p3100s1.zip › Supplementary Materials and Methods.docx]

**Supplementary Materials and Methods**

**Plasmid, virus and reagent** The pcDNA3.1-Akt1, pcDNA3.1-myr-Akt1, and pcDNA3.1-c-Myc were generated previously ^53^. The human ASCT2 promoter (-880 ~ +22) was subcloned into pGL4 vector. Lentivirus NDRG2 and ASCT2 were packaged in Hanbio Biotechnology (China). CB-839 (HY-12248) and V-9302 (HY-112683) were purchased from MedChemExpress. 2-NBDG (N13195) was from Invitrogen.

**Immunoprecipitation and Western blotting** Cells were harvest and extracted. The extracts were incubated with anti-Fbw7 antibody overnight at 4 ℃. After blended with Protein A/G Dynabeads® (Thermofisher), the complex was washed twice with PBS, in turn to be resuspended and boiled with 2 X SDS loading buffer and subjected to Western blot analysis. Western blotting assay was performed as previously described ^53^. Primary antibodies were used at dilutions of 1:200 for anti-FBW7 (sc-293423, Santa Cruz), 1:1000 for anti-c-Myc (#ab32072, Abcam), anti-p-Akt (S473) (#4051, Cell Signaling Technology), anti-Akt (#2938, Cell Signaling Technology), anti-E-cadherin (#144725, Cell Signaling Technology), anti-Vimentin (#5741, Cell Signaling Technology), anti-NDRG2 (H00057447-M03, ABNOVA), anti-ASCT2 (#ab237704, Abcam), anti-Snail (#3879, Cell Signaling Technology), anti-Slug (#9585, Cell Signaling Technology), anti-GLS1 (#ab200408, Abcam), anti-GLS2 (#ab150474, Abcam) and anti-β-actin (#A5441, Sigma).

**Protein degradation assay** MC3 cells or MEF cells were incubated with CHX (10 μg/ml, Sigma) for indicated time. Cells were then harvest and western blotting was performed as described above.

**Quantitative PCR** The RNA was extracted and cDNA was generated through GoScript Reverse Transcription System (Promega). The quantitative PCR (qPCR) assay was performed as described previously [20]. Primer sequences are available on request.

**Cell-based assays** MTT assay, transwell assay, glycolysis and glutamine relevant assays were performed as described previously ^27-29,54^.

**ASCT2 promoter related assays** Luciferase reporter assay and Chromatin immunoprecipitation were performed as described previously ^53^. The sequences of PCR primers detecting ASCT2 promoter are available on request.

**RNAseq experiments** The RNA were extracted from MEC1 cells, MC3 cells and MC3 cells with NDRG2 overexpression (n=3). Paired-end libraries were synthesized by using the TruSeq™ RNA Sample Preparation Kit (Illumina, USA) following TruSeq™ RNA Sample Preparation Guide. Briefly, the poly-A containing mRNA molecules were purified using poly-T oligo-attached magnetic beads. Following purification, the mRNA is fragmented into small pieces using divalent cations under 94 for 8 min. The cleaved RNA fragments are copied into first strand cDNA using reverse transcriptase and random primers. This is followed by second strand cDNA synthesis using DNA Polymerase I and RNase H. These cDNA fragments then go through an end repair process, the addition of a single ‘A’ base, and then ligation of the adapters. The products are then purified and enriched with PCR to create the final cDNA library. Purified libraries were quantified by Qubit® 2.0 Fluorometer (Life Technologies, USA) and validated by Agilent 2100 bioanalyzer (Agilent Technologies, USA) to confirm the insert size and calculate the mole concentration. Cluster was generated by cBot with the library diluted to 10 pM and then were sequenced on the Illumina NovaSeq 6000 (Illumina, USA).

**TCGA patient data analysis**  The Cancer Genome Atlas cBioPortal was used to determine the correlation of NDRG2 and ASCT2 in the dataset of different types of tumors. NDRG2 and ASCT2 were entered as the query genes.and co-expression was provided through the cBioPortal user interface. The raw data were downloaded from TCGA through the cBioPortal and manually graphed in Prism (Graphpad) with relative mRNA alterations. For gene expression association analysis, gene expression levels were normalized for each patient to the median expression across all patients. A log conversion was applied to the normalized, averaged NDRG2 mRNA and ASCT2 mRNA expressions and then plotted as a correlation graph in Prism software with a linear regression curve.
