# Supplementary figures and images for: NDRG2 ablation reprograms metastatic cancer cells towards glutamine dependence via the induction of ASCT2
Source: Int J Biol Sci. 2020 Oct 16;16(16):3100–15. doi: 10.7150/ijbs.48066 (PMC7645990; doi:10.7150/ijbs.48066)

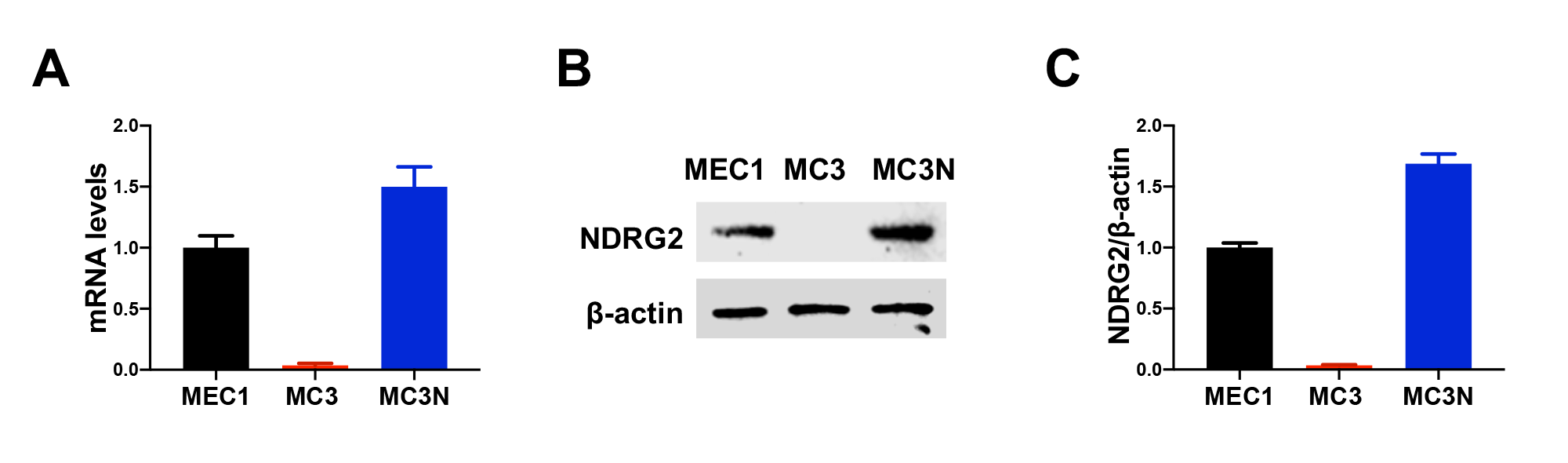

Supplement: Supplementary file 1 — Supplementary figures and tables. [file ijbsv16p3100s1.zip › Supplementary figure 1.tif]
